# Synergistic Antioxidant and Anti-Inflammatory Effects of Phenolic Acid-Conjugated Glutamine–Histidine–Glycine–Valine (QHGV) Peptides Derived from Oysters (*Crassostrea talienwhanensis*)

**DOI:** 10.3390/antiox13040447

**Published:** 2024-04-10

**Authors:** Soyun Choi, Sohee Han, Seungmi Lee, Jongmin Kim, Jinho Kim, Dong-Ku Kang

**Affiliations:** 1 Department of Chemistry, Incheon National University, Incheon 22012, Republic of Korea; yun00902@inu.ac.kr (S.C.); lsm5721@inu.ac.kr (S.L.); jinho@inu.ac.kr (J.K.); 2 WellPep Co., Ltd., Incheon 22012, Republic of Korea; soheehan0701@gmail.com (S.H.); help@wellpep.co.kr (J.K.); 3 Bioplastic Research Center, Incheon National University, Incheon 22012, Republic of Korea; 4 Research Institute of Basic Sciences, Core Research Institute, Incheon National University, Incheon 22012, Republic of Korea

**Keywords:** antioxidant, anti-inflammatory, anti-wrinkle, natural peptide, cosmetic material, phenolic acids

## Abstract

The glutamine–histidine–glycine–valine (QHGV), a peptide derived from oysters, exhibits antioxidant activity and is being actively researched as a potential pharmaceutical and functional cosmetic ingredient. In this study, we synthesized the QHGV peptide and explored the hitherto unknown anti-inflammatory effects of QHGV. The antioxidant property was also characterized by conjugating with various naturally derived phenolic acids, such as caffeic, gallic, ferulic, sinapinic, and vanillic acids. Conjugation with phenolic acids not only enhanced the antioxidant activity of QHGV but also diminished the lipopolysaccharide-induced generation of reactive oxygen species (ROS) in the murine macrophage cell line, RAW 264.7. The reduction in the levels of reactive oxygen species led to the reduced mRNA expression of inducible nitric oxide synthase *(iNos)* and cyclooxygenase 2 *(Cox-2)*, resulting in an anti-inflammatory effect through the inhibition of the phosphorylation of mitogen-activated protein kinase, including extracellular signal-activated protein kinase, c-Jun NH2-terminal kinase, and p38. Furthermore, the phenolic acid-conjugated peptides increased the mRNA and protein levels of collagen type I, indicative of a wrinkle-improvement effect. The phenolic acid conjugates of the peptide were not cytotoxic to human keratinocytes such as HaCaT cells. These results suggest that phenolic acid conjugation can enhance the potential of peptides as drug and cosmetic resources.

## 1. Introduction

The pursuit of beauty and healthy skin is a universal quest that impacts the self-expression and confidence of an individual and has cemented its significance in scientific research [1]. The identification of efficacious antiaging interventions is a fascinating and rapidly evolving area of study [2]. Among various antiaging agents, peptides have emerged as a novel class of cosmetic ingredients, with the industry seeking to maximally capitalize on their potential and multifunctionality [3]. These amino acid polymers have diverse biological functions, including the promotion of cell proliferation and anti-inflammatory, antioxidant, and anti-wrinkle benefits [4,5]. The pioneering synthesis of glutathione and the first cosmetic application of the copper-conjugated glycine–histidine–lysine (Cu-GHK) peptide in the late 1980s are landmarks in the innovative trajectory of peptide utilization [6,7]. Exhibiting high efficacy at low concentrations and high stability characteristics, peptides hold great promise owing to their potent physiological activities [8].

Among the myriad sources of bioactive peptides, marine organisms have garnered increasing attention for their physiologically active compounds. Oysters are a nutritionally dense, protein-rich, and low-fat food source, replete with an array of vitamins and minerals that have a global culinary appeal [9,10]. Global oyster production exceeded six million tons in 2018, as per the Food and Agriculture Organization of the United Nations [11]. Oysters are particularly rich in amino acids, such as taurine, glycine, and alanine, with essential amino acids representing approximately 40% of their total amino acid content [11,12]. Oyster-derived peptides display a spectrum of activities, including antioxidant, antihypertensive, antibacterial, anti-inflammatory, anti-melanogenic, anti-wrinkle, and anticancer effects [13,14,15]. The glutamine–histidine–glycine–valine (QHGV) peptide, isolated from *Crassostrea talienwhanensis*, exhibits considerable antioxidant activity, which bolsters its potential for use as a pharmaceutical and cosmetic ingredient [16].

In this study, we evaluated QHGV as a candidate for use in skin aesthetics and health, seeking to expand upon the existing knowledge of its properties. We aimed to synthesize QHGV peptides mimicking the sequence derived from oysters and conjugate them with naturally occurring phenolic acids, including caffeic acid, gallic acid, ferulic acid, sinapinic acid, and vanillic acid, to enhance their antioxidant capabilities. Phenolic acids, known as plant-derived natural antioxidants belonging to polyphenols, are recognized for their biological activity in the human body [17]. Leveraging these advantages, our objective was to develop natural multifunctional peptide materials capable of replacing artificial ingredients.

We explored the synergistic effects of this enhancement on the antioxidant, anti-inflammatory, and wrinkle-reducing efficacies of phenolic acid-conjugated QHGV peptides (PA-QHGVs). We demonstrate a previously undocumented anti-inflammatory effect of QHGV, which could be pivotal against factors such as ultraviolet radiation, diet, smoking, stress, and environmental pollutants, which accelerate skin aging by increasing reactive oxygen species (ROS) and inflammatory mediators, such as inducible nitric oxide synthase (*iNos*) and cyclooxygenase 2 (*Cox-2*) in response to stimuli, for instance, in the form of lipopolysaccharides (LPS) [18,19]. We endeavored to understand how phenolic acid conjugation to the QHGV peptide augments its antioxidative and anti-inflammatory activities to illuminate its viability as a multifunctional ingredient in the cosmetic industry.

## 2. Materials and Methods

### 2.1. Materials and Chemicals

The synthesized peptide glutamine–histidine–glycine–valine (QHGV) and PA-QHGVs were obtained from WellPep (Incheon, Republic of Korea). The molecular weights of QHGV, caffeoyl-QHGV (Ca-QHGV), galloyl-QHGV (Ga-QHGV), feruloyl-QHGV (Fe-QHGV), sinapoyl-QHGV (Si-QHGV), and vanilloyl-QHGV (Va-QHGV) were 440, 601.66, 591.62, 615.68, 645.71, and 589.65 Dalton. Dulbecco’s modified Eagle’s medium (DMEM), heat-inactivated fetal bovine serum (FBS), and antibiotic cocktail (penicillin and streptomycin) were purchased from WELGENE Inc. (Gyeongsan, Republic of Korea). Primary antibodies against β-actin and GAPDH were purchased from Santa Cruz Biotechnology Inc. (Dallas, TX, USA). The primary antibodies against COL1A1 (collagen I), ERK 1/2, phospho-ERK 1/2, JNK, phospho-JNK, p38, and phospho-p38, as well as the horseradish peroxidase-conjugated secondary antibody, were purchased from Cell Signaling Technology (Danvers, MA, USA). The RNA extraction kit was purchased from Qiagen (Venlo, The Netherlands), and the cDNA synthesis kit was obtained from TaKaRa Bio Inc. (Kusatsu, Japan). Trypsin, lipopolysaccharides (*Escherichia coli*), dexamethasone, cisplatin, 2,2′-azino-bis (3-ethylbenzothiazoline-6-sulfonic acid) diammonium salt (ABTS), The 2,2-diphenyl-1-picrylhydrazyl (DPPH), L-ascorbic acid, gallic acid, 3-(4,5-dimethylthiazoyl-2-yl)-2,5-diphenyl tetrazolium bromide (MTT), 2′,7′-Dichlorodihydrofluorescein Diacetate (DCFH-DA), and all other chemicals were purchased from Sigma-Aldrich^®^ (St Louis, MO, USA). The murine macrophage cell line (Raw 264.7) was purchased from Sigma-Aldrich^®^ (St. Louis, MO, USA), and the human keratinocyte cell line (HaCaT) was obtained from the Korean Cell Line Bank (Seoul, Republic of Korea).

### 2.2. Cell Culture

Murine macrophage (RAW 264.7) and human keratinocyte (HaCaT) cells were cultured in DMEM containing 10% (*v*/*v*) FBS and 1% (*v*/*v*) penicillin-streptomycin (10,000 IU or 10,000 μg/mL). All cells were grown at 37 °C under a 5% CO_2_ atmosphere.

### 2.3. Cell Viability Assay

Cell viability was assessed using an MTT assay. HaCaT cells (1.0 × 10^4^ cells/mL) were seeded in a 96-well plate and cultured for 24 h. Thereafter, the cells were treated with the peptide solution (0–500 µM) for 24 h. DMEM was used as a blank control, and cisplatin (0–500 µM) was used as a positive control. The MTT solution was prepared by dissolving it in phosphate-buffered saline (PBS) at a concentration of 5 mg/mL. 10 μL of the prepared MTT solution was added to each well, and the plate was incubated for 4 h. After removing the culture medium, 200 μL of dimethyl sulfoxide (DMSO) was added to each well to solubilize any deposited formazan. The absorbance at 560 nm was measured using an Epoch microplate Spectrophotometer (BioTek, Winooski, VT, USA).

### 2.4. ABTS Radical Scavenging Activity Test

The ABTS radical cation solution was prepared by mixing 1 mL of a 14 mM ABTS solution and 1 mL of 4.9 mM potassium persulfate. The mixture reacted in the dark at room temperature for 16 h. The ABTS solution was diluted with PBS (1:50, *v*/*v*), and 190 μL of it reacted with 10 μL of the sample solution in a 96-well plate at room temperature for 5 min. The absorbance at 734 nm was measured using a SpectraMax iD3 microplate reader (Molecular Devices, San Jose, CA, USA), and the ABTS radical scavenging activity rate was calculated using the following equation:

ABTS radical scavenging activity (%) = 1 − (absorbance of sample/absorbance of water) × 100

### 2.5. DPPH Radical Scavenging Activity Test

The DPPH method is based on a mechanism similar to the ABTS method. A 0.1 mM DPPH radical solution was prepared in either methanol or ethanol. In a 96-well plate, 100 μL of the DPPH solution and 100 μL of the sample were treated and incubated at room temperature in the dark for 30 min. Absorbance at 517 nm was then measured using a SpectraMax iD3 microplate reader (Molecular Devices, San Jose, CA, USA). The solvents used for the samples were methanol for peptide samples and ethanol for phenolic acid samples. The DPPH radical scavenging activity rate was calculated as follows:

DPPH radical scavenging activity (%) = 1 − (absorbance of sample/absorbance of water) × 100

### 2.6. RNA Isolation and qRT-PCR

HaCaT (3.0 × 10^5^ cells/well) and RAW 264.7 (1.5 × 10^5^ cells/well) cells were seeded in six-well plates and cultured for 24 h. The peptide and control groups were pretreated with 500 and 0 μM of the peptide solution, respectively, for 2 h, followed by stimulation with 0.1 μg/mL of LPS for 24 h. Total cellular RNA was isolated using the RNeasy Mini Kit (Qiagen, Santa Clara, CA, USA). Total RNA was used to synthesize complementary DNA (cDNA) using the PrimeScript™ 1st strand cDNA synthesis kit (TaKaRa, Kusatsu, Japan). The sequences of oligonucleotide primers for amplifying inducible nitric oxide synthase *(iNos*), cyclooxygenase-2 (*Cox-2*), and collagen type I alpha 1 chain (*Col1a1*) synthesized from Bioneer (Daejeon, Republic of Korea) and glyceraldehyde-3-phosphate dehydrogenase (*Gapdh*) synthesized from Macrogen (Seoul, Republic of Korea) are listed in Table 1. For the polymerase chain reaction (PCR), after initial denaturation at 94 °C for 5 min, amplification was performed for 40 cycles as follows: denaturation at 94 °C for 30 s, annealing at 53.7 °C for 1 min, and extension at 72 °C for 1 min for *Gapdh*; denaturation at 94 °C for 30 s, annealing at 54 °C for 1 min, and extension at 72 °C for 1 min for *iNos*; denaturation at 94 °C for 30 s, annealing at 56.9 °C for 1 min, and extension at 72 °C for 1 min for *Cox-2*, denaturation at 94 °C for 10 s, annealing at 64 °C for 15 s, and extension at 72 °C for 30 s for *Col1a1*. The mRNA levels of *iNos*, *Cox-2*, and *Col1a1* were normalized to that of *Gapdh*, which is a housekeeping gene. The mRNA levels were quantified using the 2^-ddCt^ method based on the Ct values.

### 2.7. Quantification of ROS Using DCFH-DA Fluorescence

RAW 264.7 cells (7.5 × 10^4^ cells/mL) were seeded on sterile cover slips placed in the wells of a 12-well plate and cultured for 24 h. The cells were pretreated with peptide solutions (500 μM) and carnosine (500 μM) for 2 h and stimulated with LPS (0.1 μg/mL) for 24 h. Subsequently, they were treated with a 10 μM DCFH-DA solution for 30 min and washed with PBS. The washed cells were fixed with a 4% paraformaldehyde solution for 5 min, and intracellular ROS fluorescence was observed using a DMi8 inverted microscope (Leica, Wetzlar, Germany).

### 2.8. Western Blot Assay

HaCaT (3.0 × 10^5^ cells/well) and RAW 264.7 (1.5 × 10^5^ cells/well) cells were seeded in the wells of a six-well plate. HaCaT and RAW 264.7 cells were treated with the peptide solutions (500 µM) for 24 and 2 h, respectively, and then stimulated with LPS (0.1 μg/mL) for 15 min. The cells were then washed with cold PBS and lysed in a cold lysis buffer. Cell debris was removed after centrifugation at 12,000× *g* for 30 min at 4 °C. Protein concentration was determined using the bicinchoninic acid (BCA) protein assay. The four-times-concentrated protein-denaturing buffer (1.25 M Tris-HCl (pH 6.8), glycerol, SDS, 2-mercaptoethanol, and 0.4% bromophenol blue) was added to the cell lysate and the mixture was boiled at 100 °C for 5 min. Proteins were separated by electrophoresis on 10% (for MAPKs) or 12% (for collagen) sodium dodecyl sulfate-polyacrylamide gel and electroblotted onto polyvinylidene fluoride membranes. The membranes were blocked with 5% skimmed milk in Tris-buffered saline–Tween (TBS-T) at room temperature for 1 h. After washing, the membranes were incubated overnight in respective primary antibody solutions (anti-phospho-ERK, anti-phospho-JNK, anti-phospho-p38, anti-collagen I, anti-GAPDH or anti-β-actin antibodies) at 4 °C. The membranes were washed with TBS-T and incubated with a horseradish peroxidase-conjugated secondary antibody solution for 1 h at room temperature. They were washed again with TBS-T and PBS and developed using an enhanced chemiluminescence reagent. Images of the immunoblots were scanned with an iBright™ CL1500 Imaging System (Thermo Fisher Scientific, Waltham, MA, USA). The band intensities of phospho-ERK, phospho-JNK, phospho-p38, and collagen I proteins were quantified by densitometric analysis using the ImageJ software 1.8.0 (National Institutes of Health, NIH, Bethesda, MD, USA). To normalize for protein loading, antibodies against β-actin and GAPDH were used, and phospho-ERK 1/2, phospho-JNK, and phospho-p38 phosphorylation levels were expressed relative to those of ERK, p38, and JNK, respectively. The levels of collagen I were normalized to those of GAPDH. Images of raw Western blots are shown in the Supplementary Data.

### 2.9. Statistical Analysis

Numerical data are expressed as means ± standard deviations. The experiments were performed at least in triplicate. Statistical analysis was performed using one-way analysis of variance (ANOVA) with a posteriori Bonferroni’s *t*-test using the SigmaStat 3.1 software (Systat Software, Chicago, IL, USA).

## 3. Results and Discussion

### 3.1. Chemical Structure of PA-QHGVs and Antioxidant Activity

**Figure 1:**
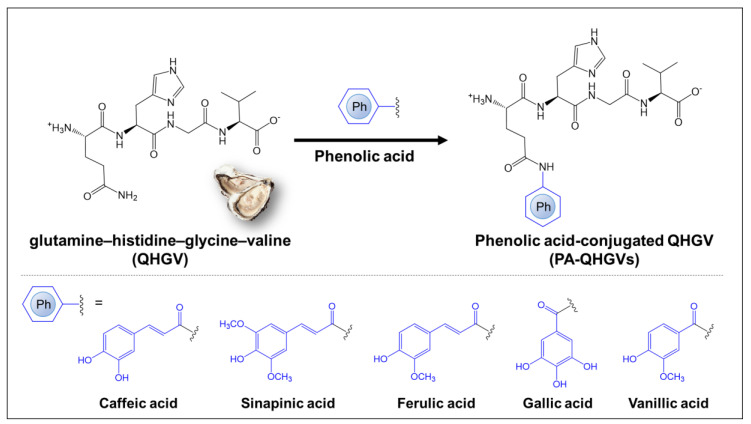
Schematic diagram for the modification of glutamine–histidine–glycine–valine (QHGV), an oyster-derived peptide, by conjugating phenolic acids. Each phenolic acid (caffeic, ferulic, sinapinic, gallic, and vanillic acid) was conjugated to the N-terminal of the QHGV peptide.

The QHGV peptide was synthesized and modified by attaching the naturally occurring phenolic acids (caffeic, gallic, ferulic, sinapinic, and vanillic acids) to the N-terminus of each peptide to enhance its antioxidant activity (Figure 1). Phenolic acids were selected based on their structural similarity to phenolic acids in which the hydrogen atom of the benzene ring was substituted with a hydroxy (-OH) or a methoxy (-OCH_3_) group. This selection was because the hydroxy and methoxy groups attached to the benzene ring affected radical cleavage and determined the antioxidant efficacy [20]. To clearly demonstrate the antioxidant effects of phenolic acids, we further validated their antioxidant abilities through the ABTS assay and DPPH assay (Figure S1). These phenolic acids can be classified into caffeic acid, ferulic acid, and sinapinic acid, which are derivatives of cinnamic acid (-CH=CHCOOH), and gallic acid and vanillic acid, derived from benzoic acid (-COOH). The number of hydroxyl groups in phenolic acids is correlated with their antioxidant capacity [21]. The presence of electron-donating or high-electron-density hydroxyl groups at the meta and para positions of gallic acid contributes to its high scavenging ability [22]. As shown in Figure S1, the hydroxyl groups in gallic acid are in the meta position relative to the carboxylic group, whereas in vanillic acid, the hydroxyl group is in the para position with respect to the carboxylic group, resulting in a lower antioxidant effect [23].

**Figure 2:**
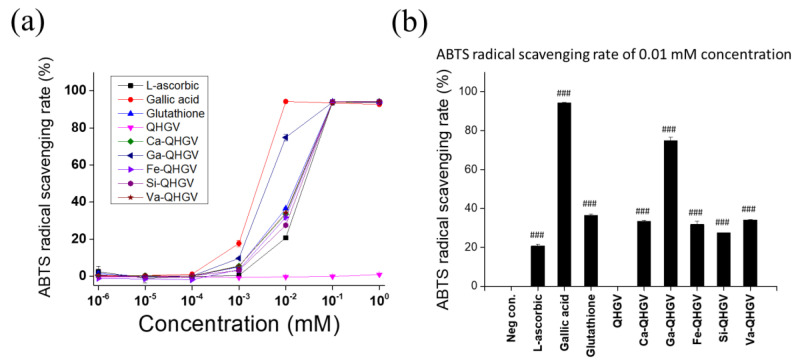
Antioxidant effect of peptides. (**a**) ABTS radical scavenging activity at various concentrations; (**b**) ABTS radical scavenging rate at 0.01 mM determined using the ABTS assay. ^###^ *p* < 0.001 compared to the untreated group (one-way ANOVA; *n* = 3).

The following phenolic acid-conjugates of QHGV were prepared: caffeoyl-QHGV (Ca-QHGV), galloyl-QHGV (Ga-QHGV), feruloyl-QHGV (Fe-QHGV), sinapoyl-QHGV (Si-QHGV), and vanilloyl-QHGV (Va-QHGV). The ABTS^•+^ radical cation assay and DPPH radical scavenging assay were used to characterize the antioxidant activity of the peptides at various concentrations between 0.000001 and 1 mM. L-ascorbic acid and gallic acid were used as positive controls to compare the antioxidant activities of the peptides. Glutathione (GSH, γ-L-glutamyl-L-cysteinyl-glycine), an antioxidant peptide used in the cosmetic industry, was also used as a positive control. The antioxidant activity was evaluated using the half-maximal inhibitory concentration (IC_50_) (Table S1). A lower IC_50_ value indicates higher antioxidant activity. With the increase in concentration, the scavenging effect of PA-QHGVs was increased. PA-QHGVs had a higher antioxidant activity than that of QHGV (Figure 2a). When the antioxidant activity of PA-QHGVs was compared with PAs, PAs indicated higher antioxidant activity than PA-QHGVs at the same concentration. Interestingly, gallic acid showed the highest antioxidant activity, and the highest antioxidant activity was also observed from Ga-QHGV among PA-QHGVs (Figure S2). However, contrary to our expectations, QHGV did not exhibit high antioxidant activity [16]. According to a previous study, peptide-based antioxidant activity progresses slowly [24]. To confirm this, we conducted experiments by synthesizing QHGV from two peptide companies. Consistent results were obtained for the three QHGV peptides, and the antioxidant activity of QHGV gradually increased over a 6 h period (Figure S3). As previously established, the antioxidant activity was apparently influenced by histidine [24]. The antioxidant effects of PA-QHGVs showed a similar trend in the DPPH assay (Figure S4). However, Va-QHGV did not exhibit antioxidant effects in the DPPH assay, which seems to be related to the weak antioxidant activity of vanillic acid observed in the DPPH assay.

The antioxidant effects of PA-QHGVs were compared at a concentration of 0.01 mM (Figure 2b). Ga-QHGV exhibited 74% radical-scavenging activity, showing the second-highest antioxidant effect after gallic acid. Ca-, Fe-, Si-, and Va-QHGVs exhibited radical scavenging activities of 33%, 31%, 27%, and 34%, respectively, which were higher than that of 20% l-ascorbic acid as the positive control. These data suggest that the conjugation of phenolic acid positively affects the antioxidant activity of QHGVs.

### 3.2. Cytotoxicity of the PA-QHGVs in HaCaT Cells

**Figure 3:**
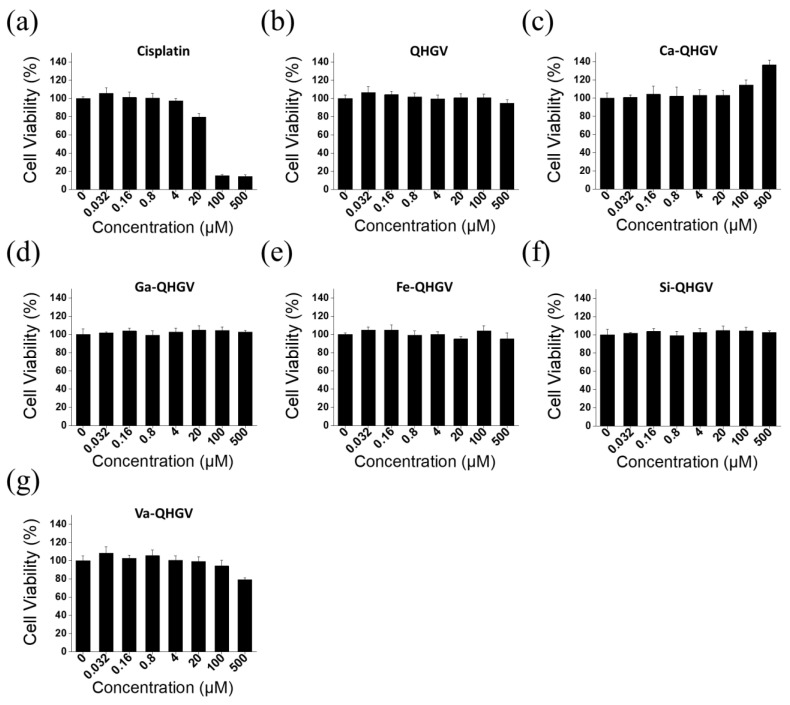
Cytotoxicity of phenolic acid-conjugated QHGV peptides (PA-QHGVs) in HaCaT cells. Cells were treated with QHGV or phenolic acid-conjugated peptides at different concentrations for 24 h.

The cytotoxicity of PA-QHGVs was evaluated using the MTT assay in HaCaT cells at various concentrations between 0.032 and 500 µM (Figure S5). Cisplatin, an anticancer drug, was used as a positive control to compare the effects of the peptides on cell viability. Cisplatin showed high cytotoxicity with cell viability of 15% at 100 μM, but the QHGV peptide showed no cytotoxicity even at 500 μM (Figure 3b). Interestingly, the Ca-QHGV peptide promoted cell proliferation, suggesting that this effect was due to the conjugation of caffeic acid, which is known to increase cell viability by protecting against the damage to DNA caused by ROS (Figure 3c) [25]. The Ga-QHGV (Figure 3d), Fe-QHGV (Figure 3e), and Si-QHGV (Figure 3f) peptides exhibited no cytotoxicity. Va-QHGV decreased the cell viability by 21% at 500 μM (Figure 3g). Thus, the attachment of phenolic acids to the peptide was not toxic to cells at the tested concentrations.

### 3.3. PA-QHGVs Attenuates LPS-Induced ROS Generation in RAW 264.7 Cells

The results of the ABTS assay (Figure 2) confirmed that the conjugation of phenolic acids enhanced the antioxidant activity of the peptides. We further investigated whether the antioxidant effect of the peptides could inhibit ROS production. In normal cells, a balance is maintained between antioxidants and ROS generated during metabolic processes [26]. However, imbalances caused by factors such as aging or other internal/external factors can lead to increased ROS levels, which, in turn, can enhance the expression of inflammation-related cytokines and trigger inflammatory responses [27].

**Figure 4:**
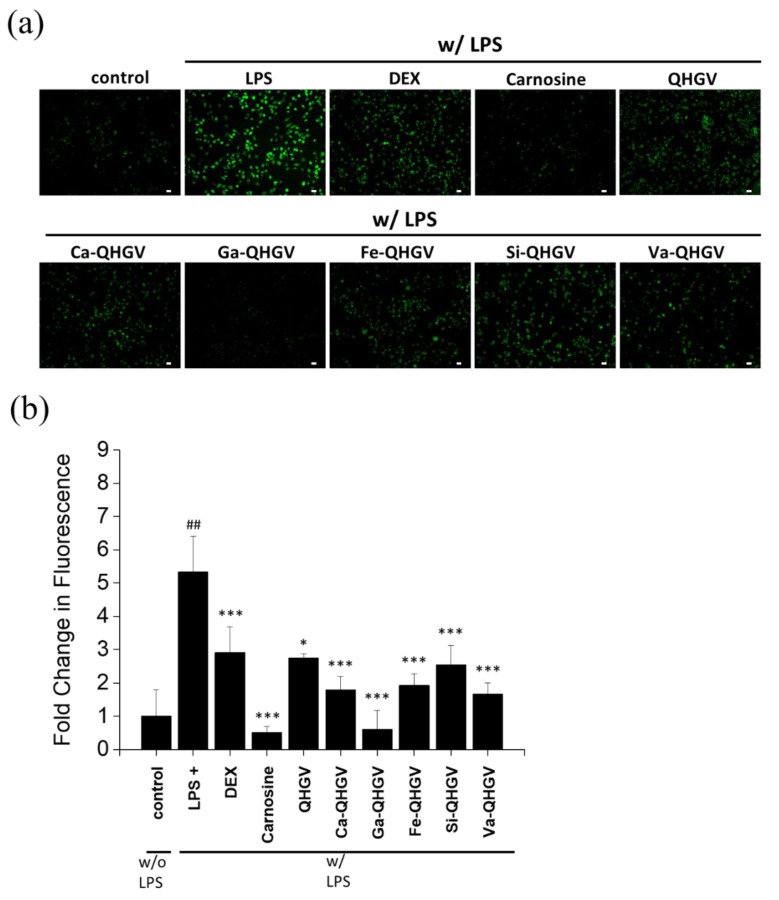
Intracellular levels of reactive oxygen species (ROS) in RAW264.7 cells. (**a**) Fluorescent microscopy images of RAW264.7 cells (scale bar = 10 μm); (**b**) Fold intensity of intracellular ROS levels in RAW264.7 cells. ^##^ *p* < 0.01 compared to the control group; * *p* < 0.05, *** *p* < 0.001 compared to the +LPS group (one-way ANOVA; *n* = 3).

Intracellular ROS levels were determined by a fluorescence microscope of cells treated with a ROS-specific fluorescent probe (DCFH-DA) (Figure 4a). The effect of the modified peptides was characterized using LPS-sensitive RAW264.7 macrophages [28]. LPS, which is derived from the outer membrane of Gram-negative bacteria, induces the first immune response by stimulating ROS [29]. Dexamethasone (DEX), a commonly used anti-inflammatory drug, and carnosine (β-alanyl-L-histidine), an anti-inflammatory peptide [30], were used as the controls. The ROS levels increased by approximately 533% in the group treated with 100 ng/mL of LPS (Figure 4b). After treatment with DEX and carnosine, the intracellular ROS levels decreased by approximately 243% and 483%, respectively, compared to that in the LPS-treated group. The decrease in intracellular ROS upon the treatment of cells with PA-QHGVs was more than the decrease experienced upon treatment with the QHGV peptide. In particular, Ga-QHGV, which showed excellent antioxidant activity (Figure 2), reduced LPS-induced ROS levels to the greatest extent. These results indicate that phenolic acid conjugation enhanced the ROS reduction efficacy of QHGV.

### 3.4. PA-QHGVs Inhibits iNos and Cox-2 mRNA Expression in LPS-Induced RAW 264.7 Cells

We hypothesized that the inhibition of ROS by PA-QHGVs affects their anti-inflammatory activity. We examined the effects of these peptides on the expression of *iNos* and *Cox-2* in LPS-induced RAW 264.7 cells [19]. *iNos*, a nitric oxide synthesizing enzyme, generates NO, and excessive NO can act as tissue-damaging molecules, triggering inflammatory responses [31,32]. Prostaglandins (PGs) generated by *Cox-2* mediate inflammatory responses. *Cox-2* is normally expressed at very low levels in healthy cells, but it is rapidly induced by inflammatory stimuli and plays a significant role [33].

**Figure 5:**
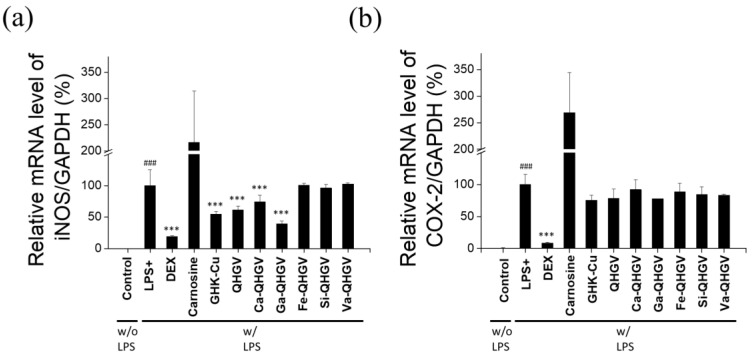
Anti-inflammatory effect on lipopolysaccharide (LPS)-induced RAW 264.7 cells. Relative mRNA levels of inducible nitric oxide synthase *(iNos*) (**a**) and cyclooxygenase-2 (*Cox-2*) (**b**) were determined using reverse transcription PCR. ^###^ *p* < 0.001 compared to the control group, *** *p* < 0.001 compared to the +LPS group (one-way ANOVA; *n* = 3).

DEX, carnosine, and an anti-inflammatory peptide, glycyl-L-histidyl-L-lysine-Cu (II) (GHK-Cu), were used as positive controls [34,35]. Data were normalized against GAPDH expression. The mRNA levels of *iNos* and *Cox-2* in RAW 264.7 cells are shown in Figure 5. LPS treatment significantly increased the expression of *iNos* and *Cox-2* compared to that in the control (no LPS). When treated with DEX, the LPS-induced increase in *iNos* and *Cox-2* mRNA levels was reduced by approximately 80% and 91%, respectively. The GHK-Cu peptide reduced the *iNos* and *Cox-2* mRNA levels by 45% and 24%, respectively. In contrast, carnosine had no significant effect, probably because the anti-inflammatory activity of carnosine is not mediated through the *iNos* and *Cox-2* pathways [36]. The QHGV peptide reduced the *iNos* and *Cox-2* mRNA levels, as did most of the PA-QHGVs peptides. Ca- and Ga-QHGVs decreased the *iNos* mRNA levels by 26% and 60%, respectively (Figure 5a). However, Fe, Si, or Va conjugation did not significantly affect the expression of *iNos* mRNA. In addition, Ca-, Ga-, Fe-, Si-, and Va-QHGVs decreased the *Cox-2* mRNA levels by 8%, 22%, 11%, 15%, and 16%, respectively (Figure 5b).

These results indicate that phenolic acid conjugates of the QHGV peptide exhibit an antioxidant effect, and this conjugation does not inhibit the anti-inflammatory effects of the QHGV peptide.

### 3.5. PA-QHGVs Antagonizes LPS-Induced Activation of the JNK/ERK/P38 Pathway

**Figure 6:**
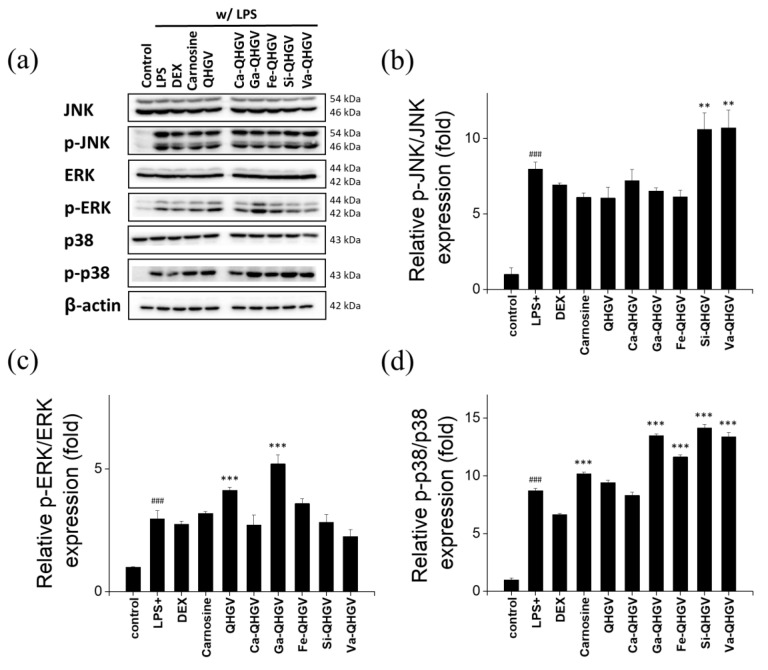
Phosphorylation of JNK, ERK, and p38 in RAW264.7 cells. (**a**) Western blot images showing phosphorylation of JNK, ERK, and p38. The fold intensity of phosphorylated JNK (**b**), ERK (**c**), and p38 (**d**) was determined by the densitometry analysis of Western blot images. Samples were derived from the same experiments and gels/blots were processed in parallel. ^###^ *p* < 0.001 compared to the control group; ** *p* < 0.01, *** *p* < 0.001 compared to the +LPS group (one-way ANOVA; *n* = 3).

To understand the mechanisms underlying the anti-inflammatory effects, we investigated the effect of PA-QHGVs on the expression of c-Jun NH2-terminal kinase (JNK), extracellular signal-activated protein kinase (ERK), and p38 using Western blotting. The proteins in the mitogen-activated protein kinase (MAPK) signaling pathways function as transcription factors that regulate proinflammatory cytokines in activated macrophages and play critical roles in inflammatory diseases [37]. The MAPK family comprises important signal molecules, including JNK, ERK, and p38. LPS treatment significantly increases the phosphorylation of JNK, ERK, and p38 compared to that in the control (no LPS) (Figure 6a). DEX markedly reduced the LPS-induced phosphorylation of JNK, ERK, and p38. p-JNK levels were decreased by 10%, 18%, and 23% in the Ca-, Ga-, and Fe-QHGV treatments, respectively (Figure 6b). p-ERK levels were decreased by 9%, 5%, and 24% in the Ca-, Si-, and Va-QHGV treatments, respectively (Figure 6c). Although most peptides did not affect the p-p38 levels, Ca-QHGV decreased it by 5% (Figure 6d).

Overall, Ca-QHGV decreased the levels of p-JNK, p-ERK, and p-p38. Ga-QHGV and Fe-QHGV decreased the levels of p-JNK, whereas Si-QHGV and Va-QHGV decreased the levels of p-ERK. These peptides exhibited anti-inflammatory effects by reducing the phosphorylation of MAPK proteins.

### 3.6. PA-QHGVs Upregulates the mRNA and Protein Levels of Collagen Type I in HaCaT Cells

ROS not only contributes to many inflammatory diseases but also reduces collagen production and causes biological changes in the components of the connective tissue matrix in the skin [38,39]. The anti-wrinkle effect mediated through the inhibition of ROS generation by PA-QHGVs was confirmed by monitoring the mRNA and protein levels of collagen type I alpha 1 chain (Collagen I) in HaCaT cells. mRNA expression was determined using RT-PCR, and protein expression was evaluated using Western blotting. The wrinkle-improving peptides used in cosmetics, acetyl hexapeptide (Argireline) and palmitoyl tripeptide-1 (PTP-1, N-(1-oxohexadecyl) glycyl-L-histidyl-L-lysine), were used as positive controls.

**Figure 7:**
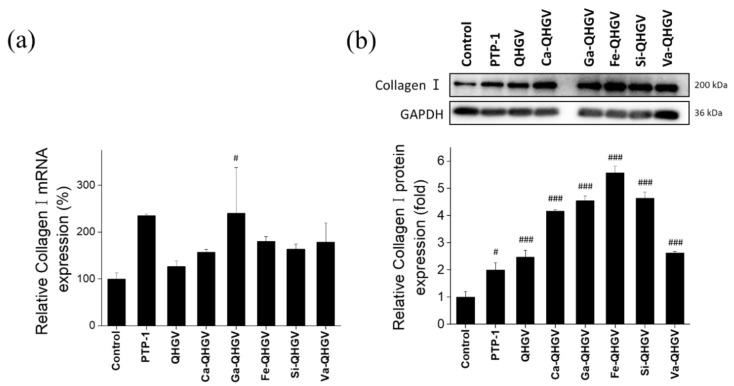
Anti-wrinkle effect of peptide on HaCaT cells. (**a**) Relative mRNA levels of collagen I (*Col1a1*) determined using reverse transcription PCR; (**b**) Relative protein levels of collagen I (COL1A1) determined using Western blot analysis. ^#^ *p* < 0.05, ^###^ *p* < 0.001 compared to the control group (one-way ANOVA; *n* = 3).

PTP-1 increased collagen I mRNA levels by 135%, whereas Argireline did not affect the expression of collagen I (Figure 7a). Argireline paralyzes nerves to exhibit wrinkle-reducing effects and does not affect the expression of collagen I [40]. In the QHGV treatment, the collagen I mRNA levels increased by 26% compared with that in the negative control group. The collagen I mRNA levels in the PA-QHGVs treatments were higher than those in the QHGV treatment. In particular, the expression of collagen I mRNA in the Ga-QHGV treatment was similar to that in the PTP-1 treatment. The Ga-QHGV peptide, which was most potent at inhibiting ROS production, increased the collagen I mRNA levels by 140%. This suggests that ROS inhibition increased the collagen I mRNA levels. Western blot results corroborated the collagen I mRNA levels (Figure 7b). QHGV and PA-QHGVs treatments increased the collagen I protein levels to a greater extent compared with the PTP-1 treatment. Fe-QHGV was the most potent, increasing the collagen I level by 457%. The levels of collagen I in the Ca-, Ga-, Si-, and Va-QHGV treatments were increased by 316%, 354%, 363%, and 163%, respectively. In contrast, QHGV only increased the levels by 147%. Thus, the conjugation of the QHGV peptide with phenolic acid significantly increased the expression of collagen I compared with that in the case of the unconjugated peptide.

## 4. Conclusions

**Figure 8:**
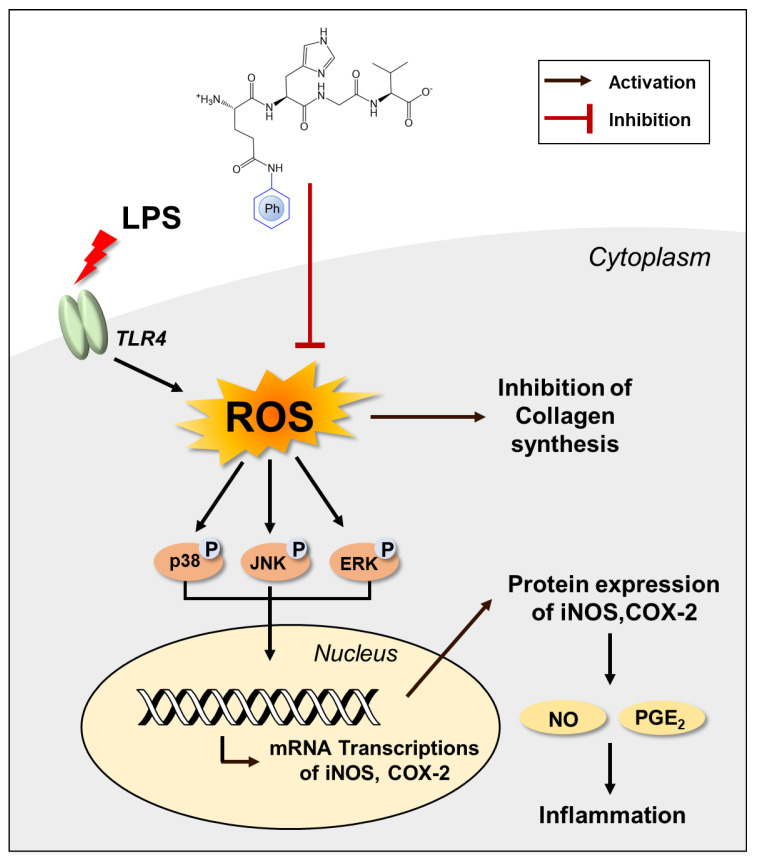
The proposed mechanism for inhibition of inflammation in RAW 264.7 and HaCaT cells by PA-QHGVs.

In this study, we unveiled the previously undisclosed anti-inflammatory activities of QHGV and enhanced its antioxidant effects by attaching phenolic acids to it. PA-QHGVs significantly reduced cellular damage by diminishing the elevated levels of reactive oxygen species (ROS) induced by LPS, demonstrating significant antioxidant effects. PA-QHGVs also decreased the mRNA expression of LPS-induced *iNos* and *Cox-2*, along with a reduction in the phosphorylation levels of JNK, ERK, and p38. This suggests that PA-QHGVs exerts its anti-inflammatory effects by inhibiting MAPK phosphorylation. Additionally, PA-QHGVs increased the mRNA and protein levels of collagen in HaCaT cells. Our data conclusively demonstrate that the anti-inflammatory and anti-wrinkle properties of PA-QHGVs stem from their antioxidant effects (Figure 8). However, whether PA-QHGVs peptides stimulate cell membrane receptors or translocate through the membrane is still unclear. Further study should be conducted to characterize whether PA-QHGVs can penetrate cells and exert its effects intracellularly.

## Figures and Tables

**Table 1:** Oligonucleotide sequences were used in the present study.

Gene Name	Direction	Nucleotide Sequence	Gene Accession Number
*iNos*	Forward	5′-GCG CCT TTG CTC ATG ACA TT-3′	NM_000625.4
	Backward	5′-ACA GGC TGC CTT GAA GGT TT-3′	
*Cox-2*	Forward	5′-AAG TTC ATC CCT GAT CCC CA-3′	NM_000963.4
	Backward	5′-GCC CTC GCT TAT GAT CTG TC-3′	
*Col1a1*	Forward	5′-CTG ACC TCC TGC GCC TGA TGT CC-3′	NM_000088.4
	Backward	5′-GTC TGG GGC ACC AAC GTC CAA GGG-3′	
*Gapdh*	Forward	5′-GAA GGT CGG AGT CAA CGG AT-3′	NM_001289745.3
	Backward	5′-TGG AAT TTG CCA TGG GTG GA-3′	

*iNos*—inducible nitric oxide synthase; *Cox-2*—cyclooxygenase-2; *Gapdh*—glyceraldehyde 3-phosphate dehydrogenase; *Col1a1*—collagen type I alpha 1 chain.

## Data Availability

The original contributions presented in this study are included in the article/Supplementary Material; further inquiries can be directed to the corresponding author.

## Supplementary Materials

The following supporting information can be downloaded at: https://www.mdpi.com/article/10.3390/antiox13040447/s1, Figure S1: Antioxidants effect of phenolic acids. (a) ABTS radical scavenging activity and (b) DPPH radical scavenging activity was characterized at various concentrations; Figure S2: Antioxidant effect of phenolic acid (PA) and PA-QHGVs peptide. ABTS radical scavenging activity was characterized at a concentration of 0.01 mM. ^###^ *p* < 0.001 compared to the untreated group (one-way ANOVA; n = 3); Figure S3: (a) Anti-oxidant effect of three different QHGV peptides from three different peptide companies. (b) The ABTS radical scavenging rate of QHGV peptides were low and slowly increased; Figure S4: Antioxidant effect of peptide. DPPH radical scavenging activity at various concentrations. Excluding Va-QHGV, PA-QHGVs effectively scavenged DPPH radicals; Figure S5: Cytotoxicity of Ca-, Ga-, Fe, Si-, and Va-QHGV at concentrations ranging from 0 to 500 μM in HaCaT cells; Table S1: IC50 Values of peptide and ABTS radical scavenging activity rank. IC50 values was calculated by GraphPad Prism 5 software.
