# Supplementary material for: Synergistic Antioxidant and Anti-Inflammatory Effects of Phenolic Acid-Conjugated Glutamine–Histidine–Glycine–Valine (QHGV) Peptides Derived from Oysters (Crassostrea talienwhanensis)
Source: Antioxidants (Basel). 2024 Apr 10;13(4):447. doi: 10.3390/antiox13040447 (PMC11047712; doi:10.3390/antiox13040447)
Supplement: Supplementary file 1 [file antioxidants-13-00447-s001.zip › antioxidants-2925312-supplementary.pdf]

Article

Synergistic Anti-oxidant and Anti-inflammatory Effects of Phenolic Acid-Conjugated Oysters (Crassostrea talienwhanensis) derived QHGV Peptide

Supplementary Materials:

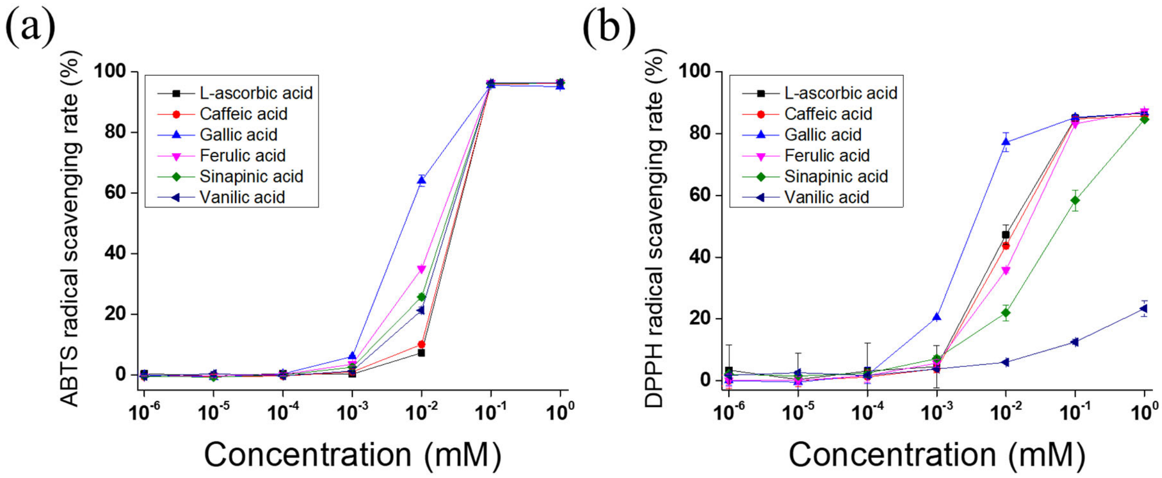

Figure S1. Antioxidants effect of phenolic acids. (a) ABTS radical scavenging activity and (b) DPPH radical scavenging activity was characterized at various concentrations.

Table S1. IC50 Values of peptide and ABTS radical scavenging activity rank. IC50 values was calculated by GraphPad Prism 5 software.

Phenolic acid	IC50 Values	Anti-oxidant Rank
Gallic acid	0.00249	1
Ga-QHGV	0.00458	2
Glutathione	0.0147	3
Va-QHGV	0.0164	4
Ca-QHGV	0.0167	5
Fe-QHGV	0.0178	6
L-ascorbic acid	0.0238	7
QHGV	-	8

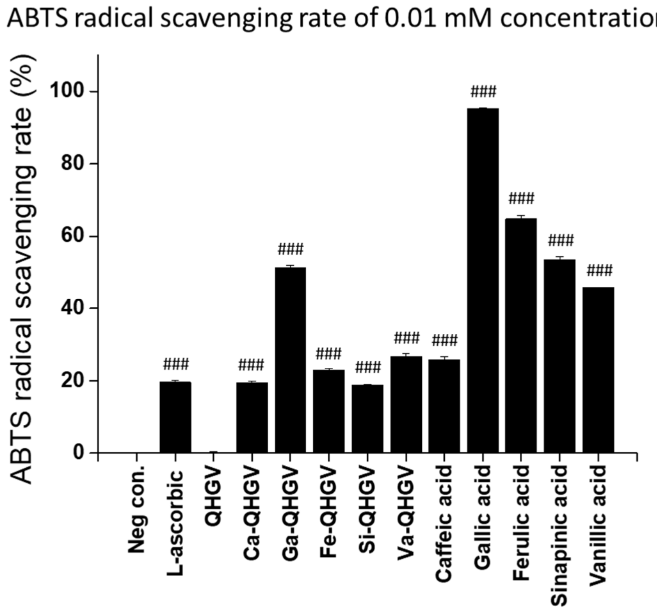

Figure S2. Antioxidant effect of phenolic acid (PA) and PA-QHGVs peptide. ABTS radical scavenging activity was characterized at a concentration of 0.01 mM. $^{###}p < 0.001$ compared to the untreated group (one-way ANOVA; n = 3).

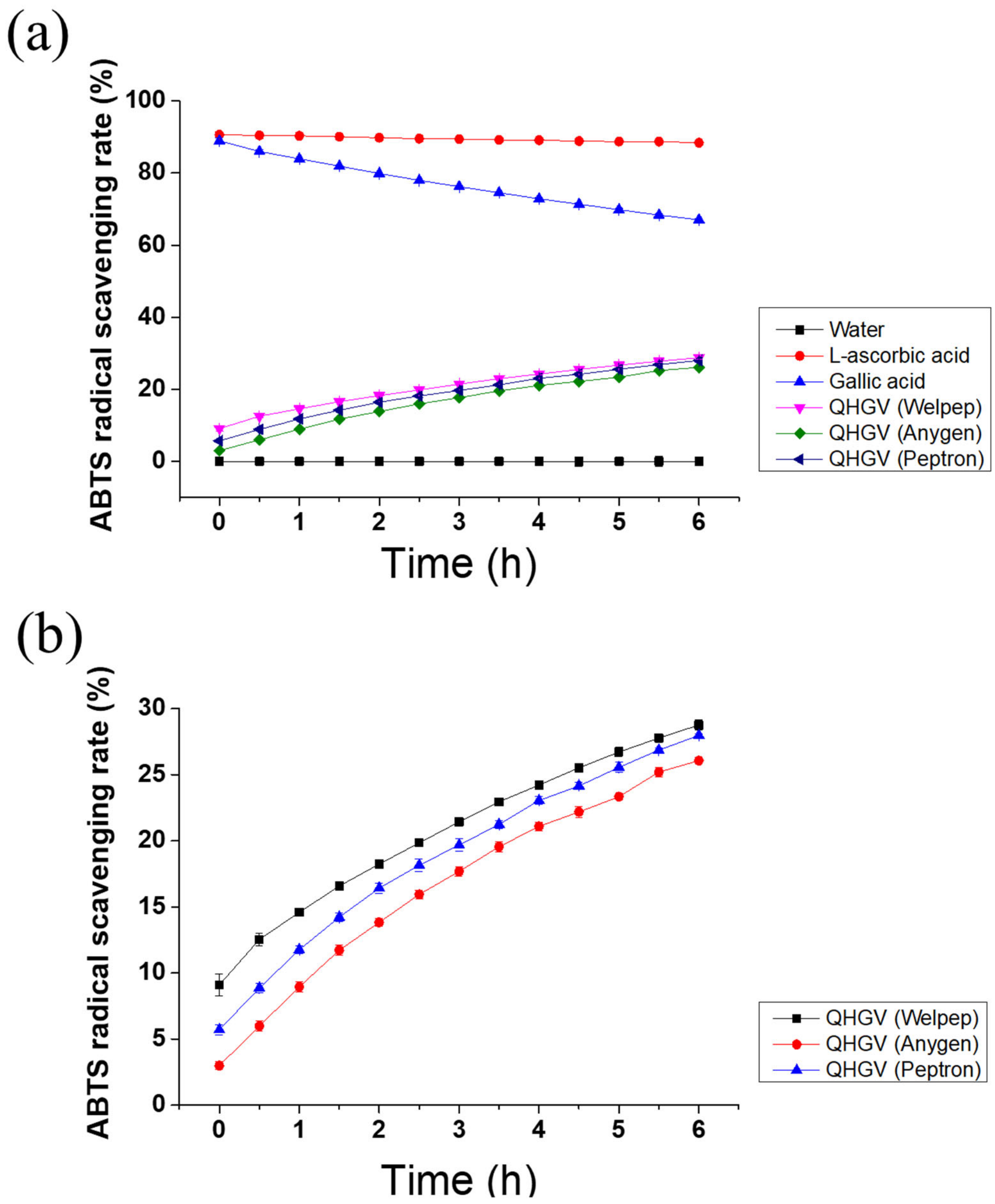

Figure S3. (a) Anti-oxidant effect of three different QHGV peptides from three different peptide companies. (b) The ABTS radical scavenging rate of QHGV peptides were low and slowly increased.

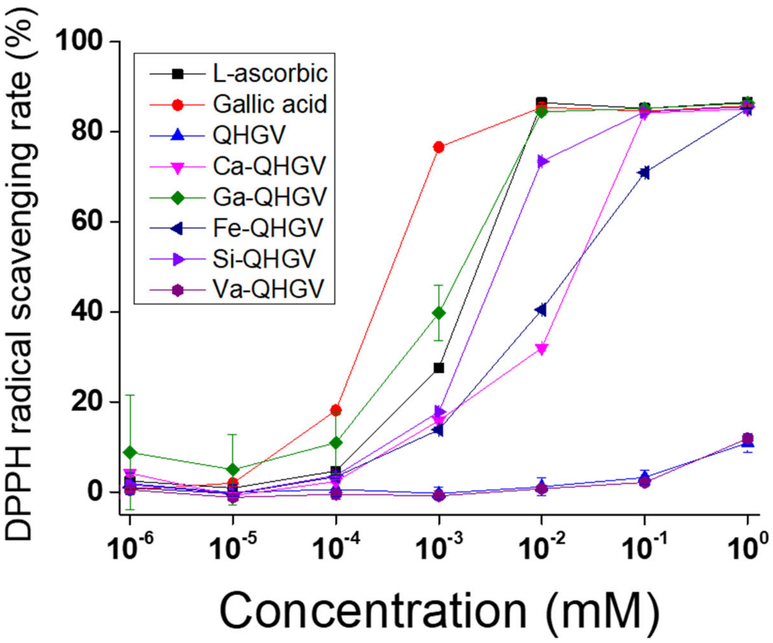

Figure S4. Antioxidant effect of peptide. DPPH radical scavenging activity at various concentrations. Excluding Va-QHGV, PA-QHGVs effectively scavenged DPPH radicals.

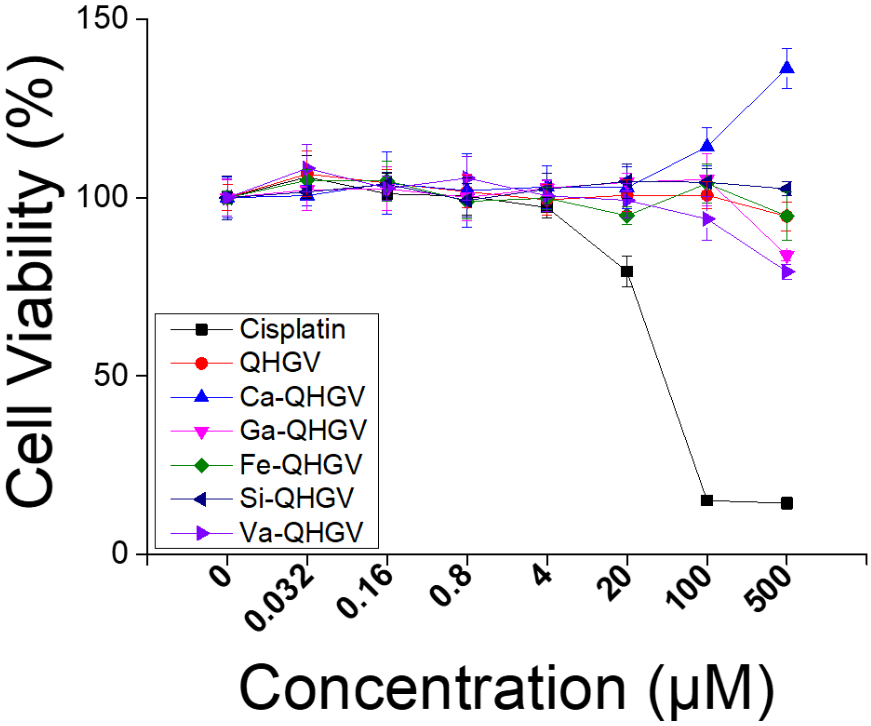

Figure S5. Cytotoxicity of Ca-, Ga-, Fe, Si-, and Va-QHGV at concentrations ranging from 0 to 500 μM in HaCaT cells.
