# Supplementary material for: Synergistic Antioxidant and Anti-Inflammatory Effects of Phenolic Acid-Conjugated Glutamine–Histidine–Glycine–Valine (QHGV) Peptides Derived from Oysters (Crassostrea talienwhanensis)
Source: Antioxidants (Basel). 2024 Apr 10;13(4):447. doi: 10.3390/antiox13040447 (PMC11047712; doi:10.3390/antiox13040447)

Western Blots raw data

Figure 6(a)
JNK
3708S,Cell signaling

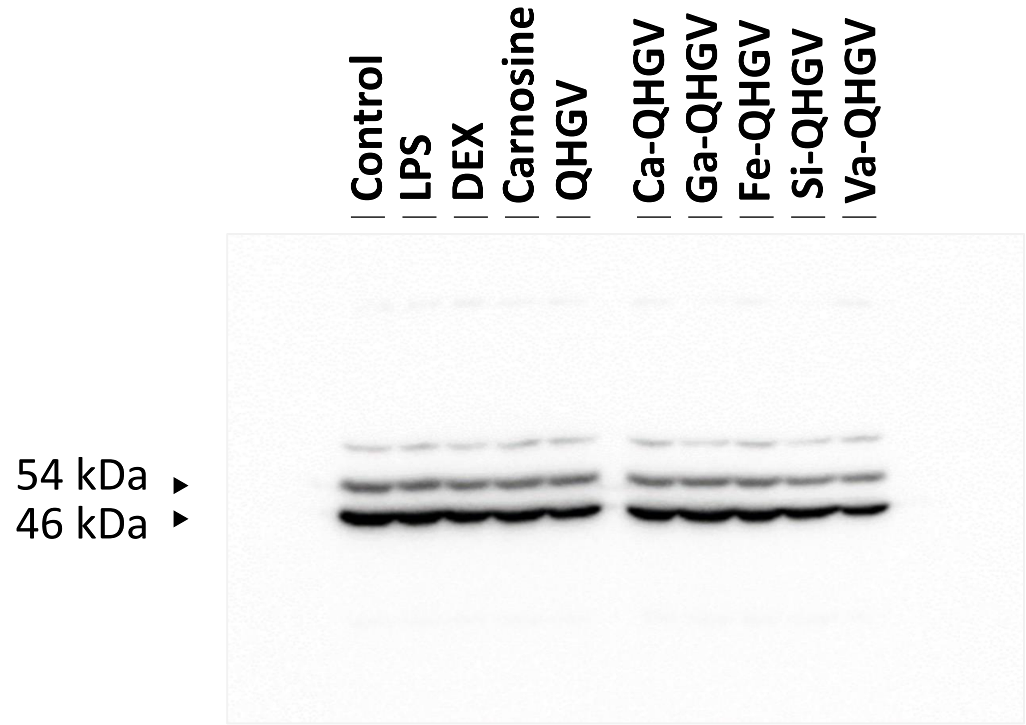

Figure 6(a)
p-JNK
9255S,Cell signaling

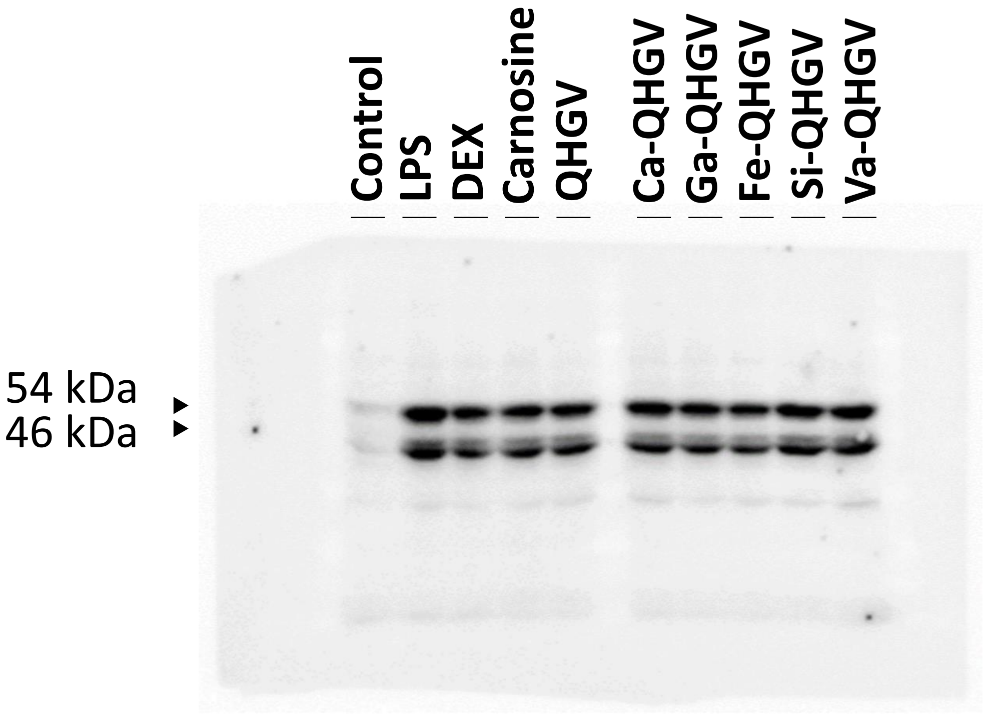

Figure 6(a)
ERK
9107S,Cell signaling

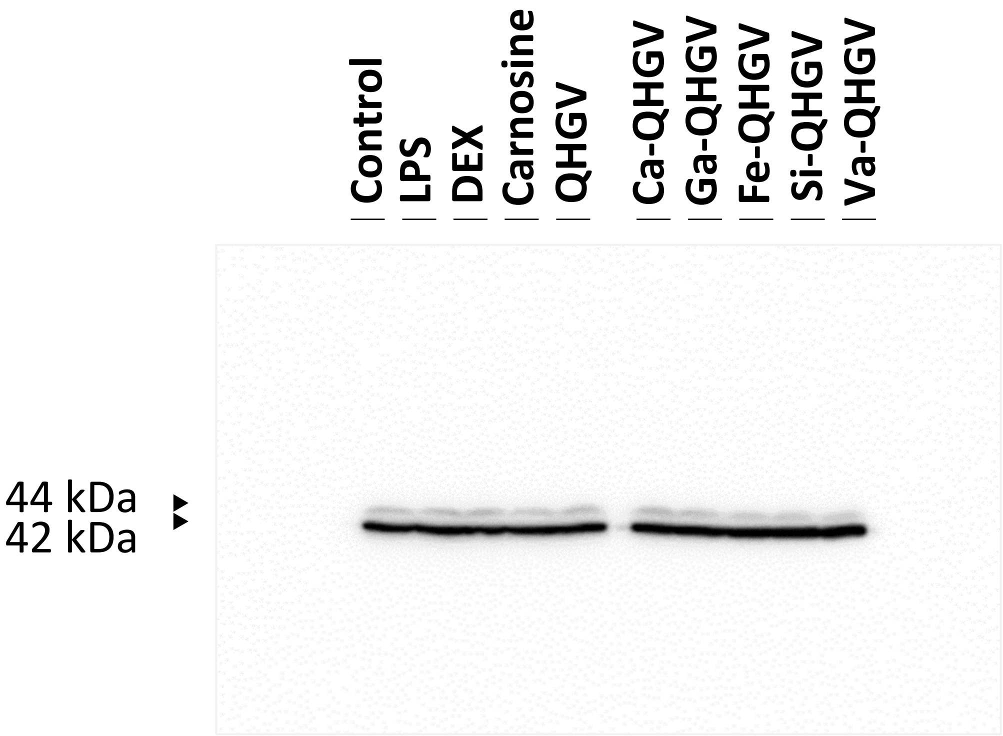

Figure 6(a)
p-ERK
9106S, Cell signaling

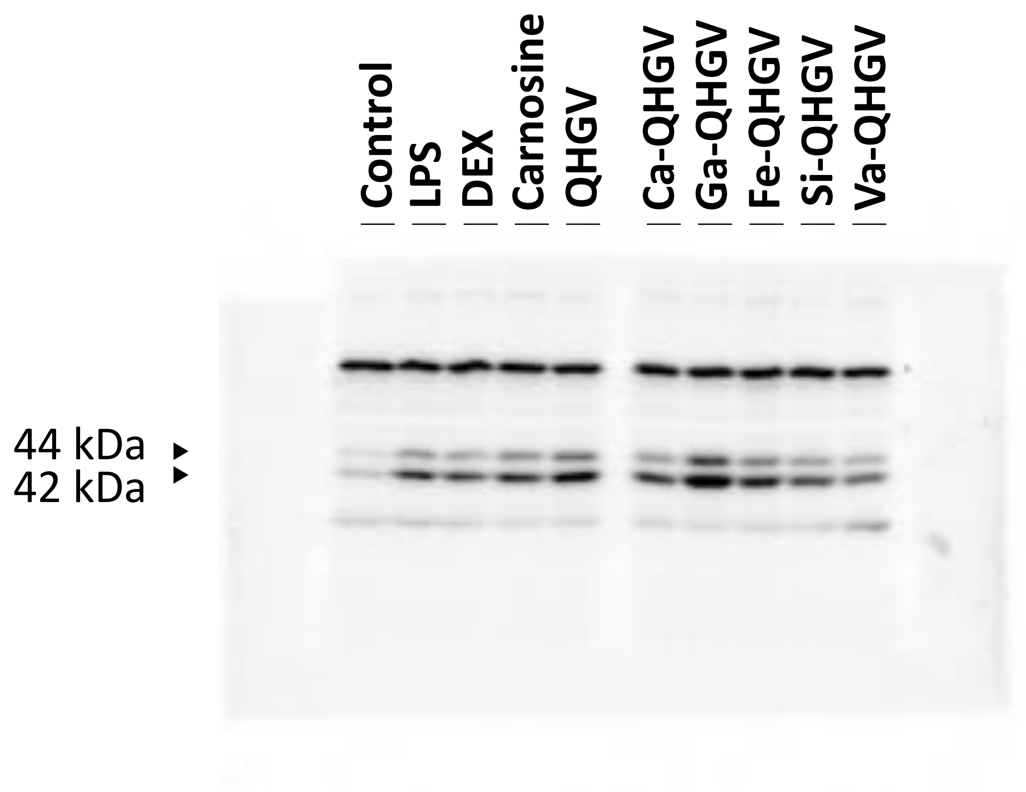

Figure 6(a)
p38
9217S, Cell signaling

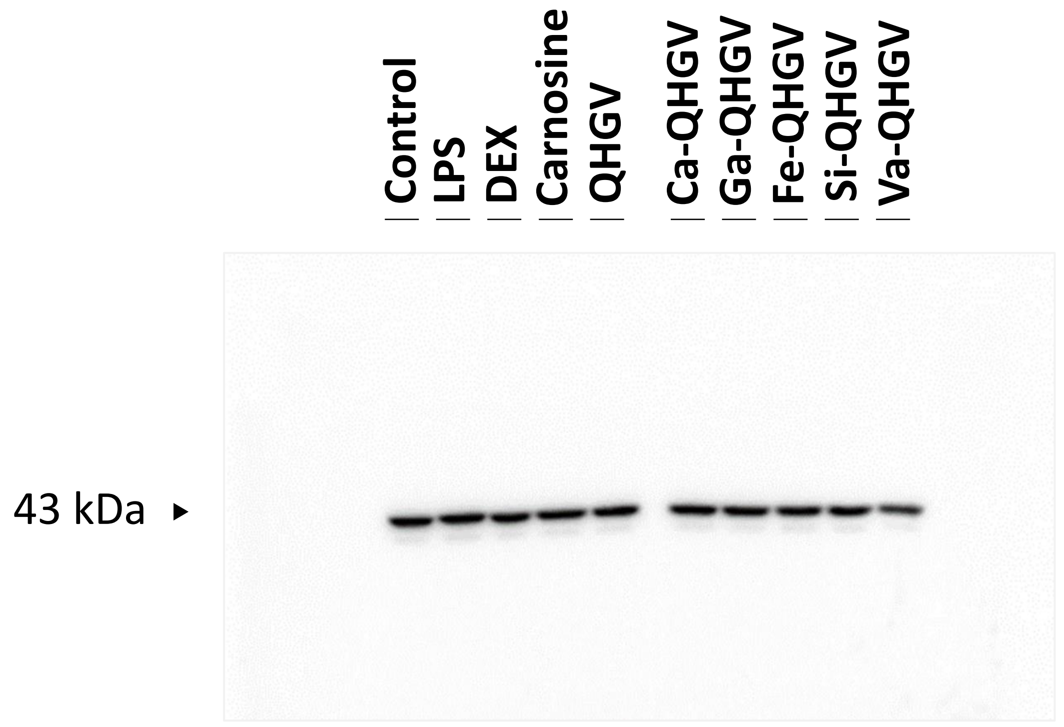

Figure 6(a)
p-p38
9216S, Cell signaling

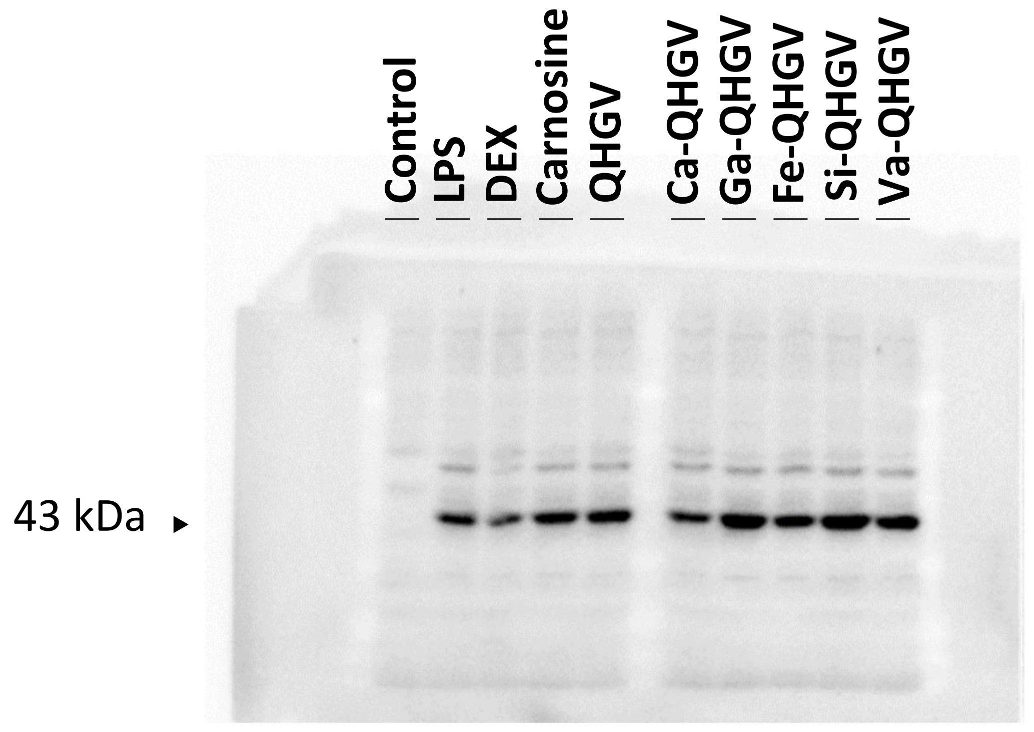

Figure 6(a)
β-actin
sc-47778, Santacruz

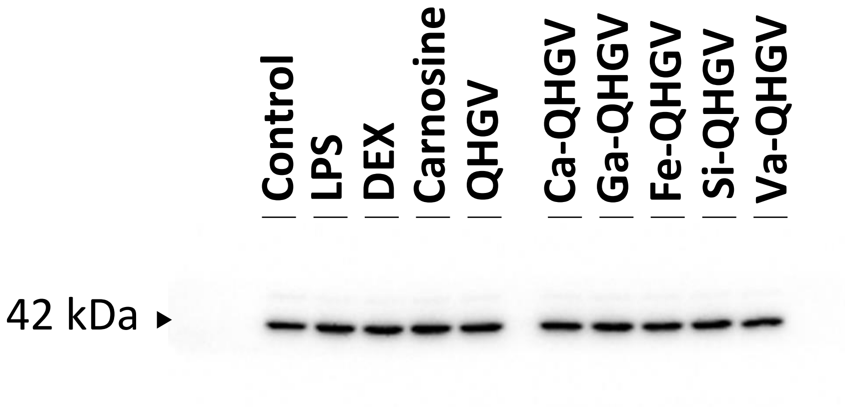

Figure 7(b)
Collagen Ⅰ
66948S, Cell signaling

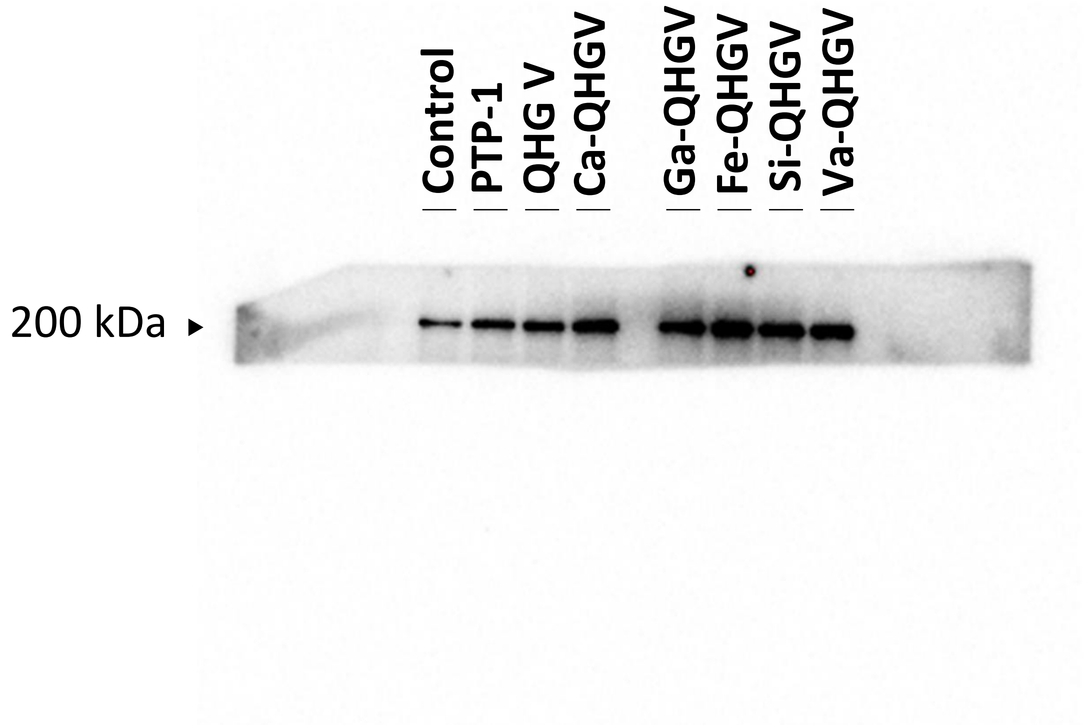

Figure 7(b)
GAPDH
sc-47724, Santacruz

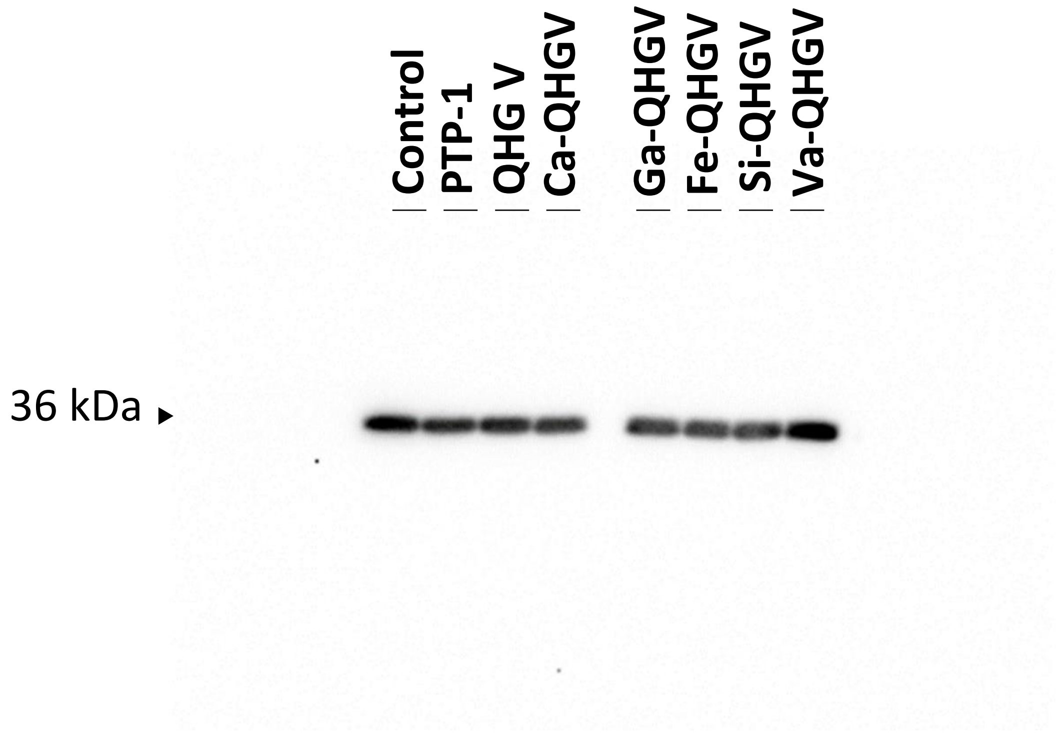

Supplement: Supplementary file 1 [file antioxidants-13-00447-s001.zip › Western Blots raw data.pdf]
